# Measuring cannabis-related knowledge, attitudes, perceptions, motivations, and influences among women of reproductive age: a scoping review

**DOI:** 10.1186/s12905-022-01673-6

**Published:** 2022-03-27

**Authors:** Kara R. Skelton, Erin Donahue, Sara E. Benjamin-Neelon

**Affiliations:** 1 Department of Health Sciences, College of Health Professions, 251 Towson Way, Towson, MD 21204 USA; 2 Department of Occupational Therapy and Occupational Science, College of Health Professions, 251 Towson Way, Towson, MD 21204 USA; 3 Department of Health, Behavior and Society, Johns Hopkins Bloomberg School of Public Health, 615 North Wolfe St, Baltimore, MD 21205 USA

**Keywords:** Marijuana, Pregnancy, Perinatal, Substance use, Psychometric properties

## Abstract

**Background:**

Cannabis use among women of reproductive age has increased substantially in recent decades. Understanding reasons for cannabis use in this population is critical for cannabis use prevention efforts. Thus, this scoping review aimed to identify and synthesize current measures on reasons for cannabis use in women of reproductive age.

**Methods:**

We searched PubMed, PyschINFO, CINAHL, and Google Scholar for relevant studies published in English between January 2010 and April 2021. Peer-reviewed, quantitative studies reporting on measures of cannabis-related knowledge, attitudes, perceptions, motivations, and influences among women of reproductive age were eligible for inclusion. We excluded studies not focused on women of reproductive age and studies reporting cannabis use prevalence data only.

**Results:**

We included 11 studies (10 primary studies and 1 review) with varying subpopulation samples of women, including non-pregnant women (n = 2), women experiencing infertility (n = 1), pregnant women (n = 4), postpartum women (n = 3), and women in the perinatal period (n = 1). Measurement topic areas included information received from health care professionals, attitudes, perceptions and experiences about cannabis use, knowledge of potential harms, and motivations for cannabis use. Most studies including measures of risk perceptions were conducted among pregnant or postpartum women (n = 4). A single study measured influences of cannabis use; no studies measured social or peer influences of use. Most studies (n = 7) created their own measures, with 2 studies using secondary data via measures from population-based surveillance systems in the United States, and one using a previously validated instrument. Recommendations for future research were centered around addressing knowledge gaps of health effects of cannabis use across different time periods, and etiology of cannabis use.

**Conclusions:**

We found vast measurement gaps in current measures of antecedents of cannabis use among women of reproductive age, providing clear direction for future research in this area. Findings necessitate psychometric evaluation of existing measures to ascertain validity and reliability, as well as development of additional measures of women’s cannabis-related attitudes, perceptions, motivations, and influences. This work is critical to guide not only epidemiologic studies, but cannabis-related prevention work as well.

**Supplementary information:**

The online version contains supplementary material available at 10.1186/s12905-022-01673-6.

## Introduction

Over the past decade, cannabis use prevalence has increased substantially across the globe [1–3]. The United Nation’s World Drug Report, 2020 estimates that in 2018, 192 million people used cannabis in the past year, equating to a global prevalence of roughly 3.9% [4]. North America, Australia and New Zealand, and West and Central Africa have substantially higher cannabis use prevalence, at 14.6%, 10.6%, and 9.3%, respectively [4]. Upticks in cannabis use prevalence are also seen among women of reproductive age, including pregnant and postpartum women [5–7]. Large increases in North America, including the United States and Canada have been seen the past decade [2, 8, 9]. In the United States, estimates of past-month cannabis use among non-pregnant women have increased from 11.0% to 2016 to 14.7% in 2019 [10]. Canada has seen a similar trend, with prevalence of cannabis use in women nearly doubling from 6.6 to 11.1% from 2004 to 2017 [11]. Yet, evidence on the etiology of cannabis use, including reasons for and influences of cannabis use among women remains largely unknown [12–14].

There is a growing body of evidence exploring women’s cannabis-related knowledge, attitudes, and perceptions of cannabis use [12, 13, 15–18]. Assessing antecedents of cannabis use among women of reproductive age throughout critical life stages (e.g., adolescence, preconception, prenatal, postpartum) is imperative for the development of tailored and effective cannabis use prevention efforts. However, a robust, in-depth assessment, including psychometric evaluation, of existing measures of these potential reasons for cannabis use in women of reproductive age has not yet been performed. Such a systematic mapping of available measures on antecedents of cannabis use would undoubtedly aid researchers and clinicians in identifying the best measure for their respective purpose and population. This evidence gap, in combination with increasing prevalence of cannabis use among women of reproductive age [2, 8, 9], supports an urgent need to examine the depth and breadth of existing instruments to measure cannabis-related knowledge, attitudes, perceptions, motivations, and influences among women of reproductive age. Further, this gap may also hinder the strength of epidemiologic studies examining women’s cannabis use.

Thus, we aimed to systematically map existing evidence on measures of cannabis-related knowledge, attitudes, perceptions, motivations, and influences among women of reproductive age, including pregnant and postpartum women. This scoping review will also serve as a necessary precursor to determine if a systematic review on this topic should be performed [19].

## Methods

This scoping review is directly aligned with the Preferred Reporting Items for Systematic Reviews and Meta-Analyses extension for Scoping Reviews (PRISMA-ScR) Checklist [20].

### Protocol and registration

We utilized the scoping review framework by Arksey and O’Malley (2005), as well as recent guidance to increase rigor and reporting of scoping reviews [19–21]. The a priori protocol for this review was drafted using the PRISMA extension for Scoping Reviews [20]. Due to the rapid nature of this review, the protocol for this review was not published, but can be accessed by contacting the authors.

### Eligibility criteria

To be included in the review, studies needed to examine or report on the development, utilization, or limitations of, measures of cannabis-related knowledge, attitudes, perceptions, motivations, and influences among women of reproductive age. Further, studies were eligible if they focused on women of reproductive age, including, but not limited to women during the preconception (12 months prior to pregnancy), prenatal (during pregnancy), and postpartum (the 12 months after birth) periods. Peer-reviewed studies written in English from any geographical location were included if they were published between 2010 and 2021. Quantitative studies were eligible; mixed-methods studies that included quantitative studies were eligible, but we extracted only quantitative information to be included in the analysis. We also included systematic reviews, with or without meta-analysis, and reviews of the literature if they included quantitative studies. We excluded studies where the population was not women of reproductive age (e.g., biological men, older adults, mixed gender populations) as well as studies that were published before 2010, published as conference abstracts or book chapters, and published in a language other than English. We also excluded studies that did not measure cannabis-related knowledge, attitudes, perceptions, motivations, or influences, as well as studies assessing and reporting on self-reported cannabis use prevalence only. Finally, we excluded reviews that included only qualitative studies.

### Information sources

To identify potentially relevant studies, we searched the following databases from January 2010 to March 2021: PubMed, PyschINFO, CINAHL, and Google Scholar. We also included the first 200 results from Google Scholar, when sorted via relevance. We limited our search from 2010 onward due to the changing nature of cannabis, including legalization, so that we captured only contemporary measures in this review. We developed the final search strategy using terms for instruments that have been previously used in systematic reviews (e.g., “questionnaire”, “instrument”, “tool”) [22], incorporating additional terms specific for our population (e.g., “women”, “prenatal”, “pregnant”, “perinatal”, “postpartum”, “breast feeding”) and topic of interest (e.g., “cannabis”, “marijuana”) [23, 24]. We piloted our search strategy for each database to ensure effectiveness in producing relevant articles. After piloting search strategies in each database, we adapted the initial search terms to exclude terms that failed to yield relevant results, which included the following terms: “survey”, “evaluation”, “assessment”, “weed”, and “CBD”. The final search strategy utilized for this scoping review is presented in Additional file 1.

### Selection of sources of evidence

We used Covidence Software, an online systematic review management tool, to streamline and manage the review process (Covidence Systematic Review Software, Veritas Health Innovation, Melbourne, Australia). As part of the import process, Covidence automatically de-duplicated citations based on a match of the citation author, title, and date. After search results were imported into Covidence, the review team performed a two-stage review process (title and abstract screening and full-text screening) to screen and identify references eligible for inclusion. Two study team members (KS and ED) piloted the screening process in Covidence on 20 citations, during which we examined both the screening process and reviewer agreement. Then, two members of the research team performed title and abstract screening independently in duplicate. Upon completion of title and abstract screening, we screened potentially relevant studies in their full-text form. We resolved disagreement between reviewers at any stage using consensus and discussion. Studies meeting all inclusion criteria moved forward for data extraction. For each included study, we carried out forward and backward citation searches to identify any potential articles not included via database searching.

### Data extraction

The research team developed a detailed data extraction form, which was piloted within Covidence. Two reviewers independently extracted study data and achieved consensus on each item. Using recommendations on relevant data fields for scoping reviews, we extracted the following data: (1) study information (e.g., author, geographic location, dates, purpose, funding); (2) population and context (e.g., study population, setting, method of recruitment); (3) measure/tool/instrument-related data (e.g., tools, measures, psychometric properties); (4) results of pilot or feasibility testing of the measure/tool/instrument; (5) limitations of the study; (6) recommendations for future research; and (7) study conclusions. Given the overall purpose of this scoping review, we did not perform quality assessment on included studies.

### Synthesis of results

Based on expected variability in how measure-related information is presented in included studies, we analyzed data both narratively and quantitatively, reporting summary of findings tables that map results in a meaningful manner. For tabular presentation of results, we first stratified by country in which the study was conducted, noting the overall sample, setting, aim, results, and conclusions of each included study. We initially intended to further stratify results by the type of measure used (e.g., knowledge, attitude, perception, motivation, influence). However, as many included studies tapped into multiple domains, this was not feasible. Additionally, we aimed to present, via tabular form and narrative synthesis, findings based on psychometric testing, differentiating between those measures for which validity and reliability have been established versus those measures that did not undergo psychometric testing. Due to the lack of psychometric testing of included measures, this was not possible. We synthesized survey characteristics in both tabular and narrative form, summarizing existing measures based on specific period(s), if any, that the measure was given (e.g., all women of reproductive age, pregnant women, breastfeeding women). We synthesized recommendations for future results and present them in tabular form.

## Results

**Fig. 1:**
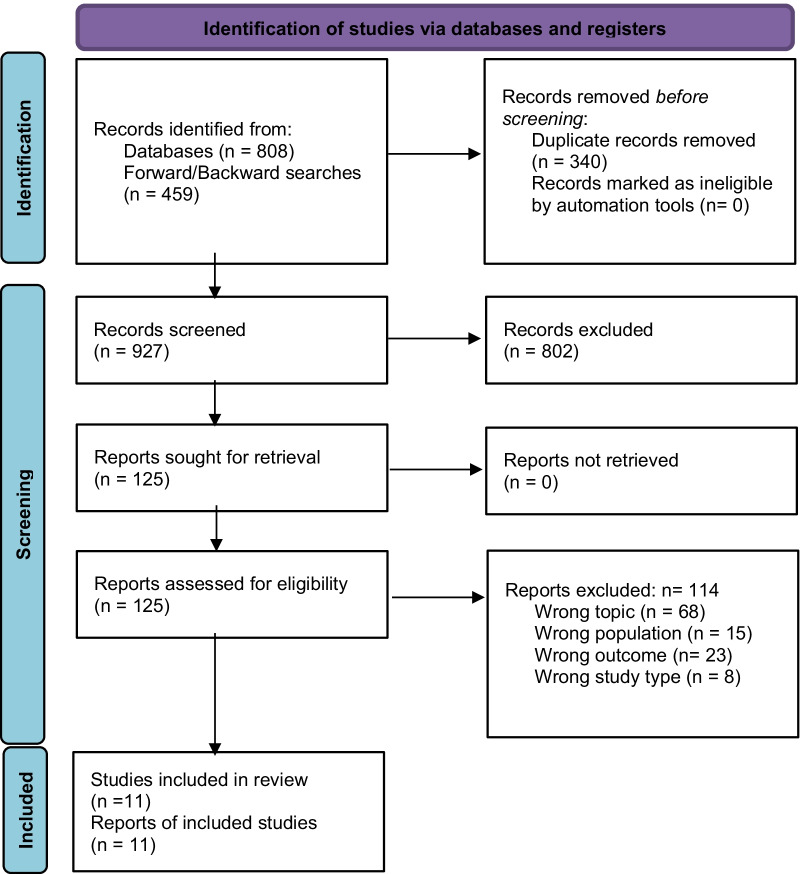
PRISMA flow diagram

Out of 927 unique citations screened, 11 studies were eligible for inclusion in this review. Figure 1 details the systematic study selection process in accordance with PRISMA guidelines.

### Key features of included studies

Table 1 describes key features of included studies, including study sample, setting, aim, results, and conclusions. We identified one systematic review [25] and 10 original studies [12–14, 26–32]. Of included studies, 3 were conducted in Canada [13, 26, 27] and 8 were conducted in the United States [14, 25, 28–32]. About 81% of studies (n = 9) were published in the past 4 years [12, 13, 25–28, 30–32]. Studies included samples of women in various life stages; two studies included non-pregnant women only [29, 32] and one included women experiencing infertility only [27]. A majority of studies included women in the perinatal period (n = 8), with some studies specifically focusing on pregnant women (n = 4) [12, 13, 28, 30] or postpartum women (n = 3) [14, 26, 31]. Most studies (n = 8) recruited women from clinics or hospital settings [12–14, 26, 28–30, 32], with 2 studies performing secondary data analyses reporting data from national surveillance systems in the United States [30, 31].

**Table 1:** Characteristics of included studies (N = 11)

Authors	Sample, setting	Aim of study	Results	Conclusions
Canada				
Bartlett 2020	478 pregnant women; Obstetrical, midwifery, and family practice clinics, greater Hamilton, Ontario area	Estimate prenatal cannabis use prevalence; evaluate pregnant women’s beliefs about transmission of cannabis during pregnancy and breastfeeding; and examine association between information from a health care provider and decision to discontinue cannabis use	Majority believed cannabis could be passed to the baby during pregnancy (94.3%) and while breastfeed (91.2%). About 23% received information about cannabis from a health care provider, while the most reported source of cannabis related information was the internet (75.1%). Receiving information from a health care provider was significantly related to continuation of prenatal cannabis use. Women continuing cannabis use during pregnancy were more likely to report receiving cannabis information from a health care provider (52%) than those who discontinued use (35%) (p = 0.035).	Education regarding prenatal cannabis use must take a nonjudgmental approach to ensure that all women are making informed decisions and adequate support is given to those with difficulty discontinuing use.
Bayrampour 2019	6 studies (5 quantitative; 1 qualitative); Integrative review	Identify and synthesize current evidence to answer the question: What are women’s perspectives on the health aspects of cannabis use, and how do these perceptions influence decision-making about cannabis use during the perinatal period?	All studies examined women’s perceptions during pregnancy, with half using a nationally representative sample. 3 articles asked: “How much do people risk harming themselves physically and in other ways when they smoke marijuana once or twice a week?” Decreasing risk perceptions of cannabis use and increasing social acceptability were seen; 70% of past-year cannabis users perceived no or slight risk of harm from using cannabis. Health care provider communications about cannabis often focused on child welfare agencies’ potential involvement and legal consequences.	Perceived safety of cannabis use, both generally and during pregnancy is increasing. Women’s perceptions of health risks due to cannabis use during the perinatal period are an important factor when making decisions about cannabis cessation, particularly as legal concerns diminish.
Jordan 2020	270 infertility patients; Mount Sinai Fertility Clinic, Toronto	Report prevalence of cannabis use among infertility patients and perception of effects of cannabis on pregnancy and fertility, cessation of use due to infertility and personal history of disclosing use to health care providers.	49% of respondents had never used cannabis and 13% reporting past-year use. Of past year users, 72% said they had or would disclose use to their healthcare provider, but only 9.4% reported their health care providers had instructed them to discontinue use. Across four measures of fertility and pregnancy health, past-year cannabis users perceived less of a negative effect compared to > 1-year users, and never users.	Cannabis use is common among infertility patients (13%). Most patients were willing to disclose cannabis use to a health care provider, but only 9.4% were counselled to discontinue use. Perceptions of negative effects of cannabis on fertility and pregnancy are correlated.
Postonogova 2020	132 postpartum women; Royal Victoria Hospital, a tertiary care obstetric center, Montreal, Quebec	Survey women who had recently given birth about their attitudes and experiences regarding the use of marijuana for the medical treatment of pain during labor	34% reported possible consideration of marijuana use for labor pain, with 25% reporting previous use during labor. Most women (52%) reported being highly or extremely worried about the effect of marijuana on the baby. Yet, most women (60%) indicated a lack of knowledge of the side effects of marijuana in labor. Nearly 60% of women reported they would be comfortable talking with their obstetrician about marijuana, with 14% reporting they would not feel comfortable discussing use with anyone. Women who would consider marijuana for labor pain were less likely to be worried about any side effect, more likely to have used marijuana for pain in the past, to agree that marijuana was an effective pain reliever.	One-third of women would consider the use of marijuana for labor pain, although there are many who are unsure of its effects. Most women would feel comfortable discussing this with their obstetrician.
United States				
Beatty 2012	50 postpartum women; large urban hospital, Michigan	Measure perceived risks and costs of marijuana and tobacco use during pregnancy	Only 1 participant selected marijuana as the substance most likely to harm the baby if used during pregnancy. When asked to rate the level of danger of marijuana to a fetus, marijuana was rated as highly dangerous with a mean rating of 9.46 (SD = 2.04). Most women indicated there is no safe amount of prenatal marijuana use (86%). 76% of women reported knowing people who felt marijuana use during pregnancy was not very dangerous for the baby. 44% of women felt that marijuana costs less to use during pregnancy than cigarettes. When asked how much women who use marijuana per day spend, participants median response was $6.00 to $10.00.	Marijuana use may be as or more prevalent than tobacco use among low-income, African American pregnant women. A broader public health response to prenatal marijuana use, is needed.
Coy 2021	4604 women with a recent live birth; PRAMS; Alaska, Illinois, Maine, New Mexico, New York, Pennsylvania, West Virginia	Describe characteristics of postpartum marijuana users; evaluate the relationship between postpartum marijuana use and breastfeeding behaviors; and assess, among postpartum marijuana users, how safety perceptions are associated with breastfeeding behaviors	Among women who used marijuana postpartum, 46.5% of reported currently breastfeeding. 91.8% believed it was unsafe to breastfeed while using marijuana; 63.0% were asked by a health care provider about marijuana use. No significant difference in breastfeeding initiation or duration between participants who used and did not use marijuana postpartum. Postpartum marijuana users who believed it was unsafe for breastfeeding women to use marijuana were significantly less likely to have initiated breastfeeding (29.9% vs.12.2%) or to have breastfed > 12 weeks (19.3% vs. 54.2%). Information delivered by health care providers to postpartum marijuana users varied; 2 in 5 were advised against breastfeeding while using marijuana, 1 in 2 were advised against marijuana use, and 1 in 15 received a recommendation to use marijuana.	Safety perceptions of marijuana use while breastfeeding may influence breastfeeding behaviors among postpartum users. Provider education about current clinical guidance of postpartum marijuana use should be considered.
Hayaki 2010	332 women; Local primary care clinics, college campuses, community health centers, and community businesses	Examine associations between endorsement of marijuana use expectancies and marijuana use frequency and severity in young females	On average participants had used marijuana “regularly” for 3.9 (± 2.6) years. Participants met an average of 2.9 (± 2.6) current marijuana abuse/dependence criteria; 70 (21.1%) met no criteria. 175 (52.7%) met current DSM IV criteria for marijuana abuse and 131 (39.6%) for marijuana dependence; 125 (37.8%) participants did not meet diagnostic criteria for either abuse or dependence. Marijuana use frequency was moderately correlated with marijuana use severity (r = 0.50, p < 0.05). Marijuana use severity was positively associated with all six expectancy subscales; the strongest were with Relaxation/ Tension Reduction (r = 0.28) and Global Negative Effects (r = 0.29).	Marijuana use expectancies may represent a clinical target that could be incorporated into future interventions.
Lynn 2019	373 women; single, academic, obstetrics and gynecology practice	Determine how women perceive the sexual experience (sexual satisfaction, sex drive, orgasm, dyspareunia, and lubrication) when using marijuana before sex and examine the effect of frequency of marijuana use on satisfaction across different sexual function domains.	Of the 176 marijuana users, 34.1% used marijuana before sex. Among users, 68.5% reported a more pleasurable overall sexual experience, 60.6% noted an increase in sex drive, and 52.8% reported an increase in satisfying orgasms. Most women reported their sexual experiences as “always to sometimes” positive, with a decrease in pain but no change in lubrication. Women who used marijuana before sexual activity were more likely to report satisfactory orgasms (aOR = 2.13; 95% CI = 1.05, 4.35) than those who reported not using marijuana. Women with frequent marijuana use, regardless of use before sex or not, were more likely to report satisfactory orgasms than those with infrequent use (aOR= 2.10; 95% CI = 1.01, 4.44).	Timing appears to be important with those who use before sex reporting a positive effect on orgasm. Among any users, the most perceived improvement was in overall experience, sex drive, orgasm, and pain. A better understanding of the role of the endocannabinoid system in women is needed, as it could help lead to development of treatments for female sexual dysfunction.
Mark 2017	306 pregnant women; Outpatient Obstetrics and Gynecology Clinics at the University of Maryland Medical Center, Baltimore MD	Investigate the relationship between current cannabis use among pregnant women and intended patterns of use and their views of cannabis legalization, knowledge of potential harms, and motivations for cessation during and after pregnancy	35% of women reported current cannabis use at the time of diagnosis of pregnancy; 34% continued use. A majority of women believed that cannabis use during pregnancy can be harmful and that it should be legalized in some form (70% and 59%, respectively. 10% reported they would use cannabis more during pregnancy if it were legalized. Those that continued use during pregnancy were less likely than those who quit to believe that cannabis use could be harmful during pregnancy (26% vs. 75%, p < 0.001). The most common motivation for cannabis cessation while pregnant was to avoid being a bad example (74%). Only 27% of women listed a doctor’s recommendation as a motivation to quit.	A significant minority of women continue to use cannabis during pregnancy, and some even increase use. Motivations to quit use during pregnancy are varied but many are related to legality.
Ng 2020	843 pregnant women; Regional perinatal center, Central New Jersey	Evaluate pregnant women’s knowledge and opinions about marijuana use, potential risks, and legalization	Most women (71%) supported or were neutral about marijuana legalization, with rates of “neutral” answers being substantially higher for prior tobacco smokers compared to nonsmokers (14.7% vs. 8.4%). Over 90% of pregnant subjects noted that they would be more likely to use marijuana in pregnancy if it were legalized. Compared to patients with prior tobacco use, nonsmokers had higher likelihood of agreeing that marijuana use during pregnancy may result in greater risks. Participants who attended high school or less were less knowledgeable about the possible risks of marijuana in pregnancy.	Pregnant women’s attitudes about marijuana use may be impacted by cannabis legalization. Pregnant women demonstrated poor knowledge about the potential risks associated with marijuana use in pregnancy.
Odom 2020	2247 pregnant women; NSDUH	Identify correlates of low risk perceptions of weekly cannabis use, past 30-day cannabis use, and frequency of cannabis use in the past 30 days among a pregnant women aged 14–44	21.6% of pregnant women did not perceive any risk with weekly cannabis use, 5.3% used cannabis in the past 30 days, and among past-month users, the average number of days of use was 15.6. Younger maternal age, early trimester of pregnancy, living below the poverty line, and co-use of tobacco and/or alcohol were all associated with not perceiving any risk associated with weekly or past 30-day cannabis use, and cannabis use in the past 30 days.	This study confirms reductions in risk perceptions of weekly cannabis use over time, increases in past 30-day cannabis use, and increases in days used, as well as identifying specific population subgroups more at risk for increases in past 30-day cannabis use.

### Measure characteristics

Characteristics of measures, including measurement domain(s), recruitment methods, population, administration modality, and a brief description of the instrument are presented in Table 2. Measurement domains among included studies were perceptions (n = 8) [13, 14, 25, 27–32], knowledge (n = 3) [12, 25, 26], attitudes (n = 2) [26, 28], intentions [12], and motivations [12]. More specifically, studies aimed to measure how information received from health care providers influenced cannabis-related decision making [13], attitudes and experiences about using cannabis during childbirth or labor [26], perceptions of cannabis use on infertility [27], risk perceptions of cannabis use [14, 28, 30], negative expectancies associated with cannabis use [29], perceptions of cannabis use and the sexual experience [32], views on cannabis legalization [12, 28], potential influence of legalization on cannabis use [12], knowledge of potential harms [12, 28], and motivations for cannabis cessation [12]. Of studies examining risk perceptions of cannabis use, 4 examined perceptions associated with prenatal cannabis use [12, 14, 28, 30], and one examined risk perceptions among postpartum, breastfeeding women [31].

**Table 2:** Measure characteristics among included studies (N = 11)

Study	Domain	Recruitment setting	Population description	Survey modality	Survey description
Canada					
Bartlett 2020	Perceptions	Clinic patients at obstetric, midwifery, and primary care practices	English-speaking, pregnant women 19-44 years of age	Online	15 questions regarding lifetime and prenatal cannabis use, perception of cannabis transmission to fetus, and resources for obtaining cannabis-related information
Jordan 2020	Perceptions	Infertility clinic patients	Women with infertility attending the Mount Sinai Fertility Clinic, read English	Written	33-question regarding cannabis use, perceptions of cannabis effects on fertility and pregnancy, cessation of use due to infertility and previous disclosure of use to health care providers
Postonogova 2020	Knowledge, attitudes	University hospital patients who had recently given birth	Women who had vaginal deliveries at the Royal University Hospital	Written	Questions assessed satisfaction with labor analgesia received, prior experiences with and knowledge of medical marijuana, attitudes towards non-medical use of marijuana, and attitudes and concerns about medical use of marijuana during labor
United States					
Bayrampour 2019	Knowledge, perceptions	Integrative Review (EDLINE, PsycINFO, EMBASE, and CINAHL)	Quantitative and qualitative studies investigating pregnant or postpartum women’s knowledge, perceptions, and perspectives about cannabis use	N/A	N/A (Review)
Beatty 2012	Perceptions	Hospital patients in a large urban hospital, shortly after giving birth, and after having slept.	Women ≥ 18 years of age, did not receive narcotic pain medication postpartum, and reported one or more in the month prior to pregnancy: illicit drug use, binge drinking, or smoking ≥ 10 cigarettes/day	Online	15 items examining risk perceptions and costs of prenatal marijuana and tobacco use, including ordering the “most harmful” substance during pregnancy, perceptions of safe amounts of use, and peer risk perceptions. Perceived financial costs, including most expensive substance, and how amount spent daily on these substances.
Coy 2021	Perceptions	Pregnancy Risk Assessment Monitoring System (PRAMS) Marijuana Supplement	Women with a recent live birth and complete information about postpartum marijuana safety perceptions and use, breastfeeding initiation, and duration. Infants were ≥ 12 weeks old at time of survey; annual response rate threshold of 55%	Written; follow-up: phone	PRAMS Marijuana Supplement, which includes questions about marijuana use, safety perceptions, and prenatal care provider’s screening and recommendation of marijuana use. Safety perceptions were assessed by the following question: “How long do you think it is necessary for a woman to wait after using marijuana to breastfeed her baby?
Hayaki 2010	Perceptions	Primary care clinics, college campuses, community health centers, and businesses	English speaking, non-pregnant women 18–24 years of age who were not seeking treatment for marijuana use but had at least monthly marijuana use in the past 3 months and had sexual activity in the past 3 months.	Interview/verbal	Marijuana use severity and associated negative consequences on the Substance Use Disorders section of the Structured Clinical Interview (SCID-I). Marijuana Effect Expectancies Questionnaire, which assesses 6 expectancy domains: relaxation/tension reduction; perceptual/cognitive enhancement; craving/physical effects; and global negative effects.
Lynn 2019	Perceptions	Clinic patients	Women ≥ 18 years of age, presenting for gynecologic care	Written	Sexual Health Survey (developed for this study). Questions about marijuana were embedded deep into the questionnaire. Frequency of marijuana use was dichotomized into frequent (once a week, several times a day) and infrequent (several times a year, once a year).
Mark 2017	Motivations, Intentions, knowledge	Clinic patients	Pregnant women who could read English	Written	26 items on frequency and quantity of cannabis use before and during pregnancy, intentions to use during the remainder of their pregnancy and postpartum, knowledge of harms (prenatal), and possible motivations to quit (for those indicating cessation or intention to quit). Motivations to quit items were modified from Chauchard et al. (2013) to apply to pregnancy. Cannabis use intentions if it were to be legalized were modeled after Fetherston and Lenton’s (2005) survey.
Ng 2020	Attitudes, Perceptions	Clinic patients	Pregnant women from various practices (a mix of commercial insured, Medicaid, and uninsured patients), presenting for prenatal or ultrasound visit	Written	6 questions about current and prior tobacco and marijuana use, including prenatal use, and frequency of use; 5 questions regarding marijuana legalization (support for recreational and medical legalization, how legalization may impact use); 5 questions queried perceptions about risks, including potential harm to pregnancy, growth restriction, preterm birth, and learning disabilities.
Odom 2020	Perceptions	Women surveyed in the 2015-2017 National Survey on Drug Use and Health (NSDUH)	Pregnant women 14–44 years of age at the time of survey	Online	Perceived Risk of weekly cannabis use was defined by recoding the original variable “perceived risk of smoking cannabis once or twice a week” into a binary variable (no risk/ do not know of any risks vs. any risk). Past 30-day cannabis use and frequency of past 30-day cannabis via mean number of days of use.

### Psychometric evaluation

Most studies created their own survey on women’s cannabis-related knowledge, attitudes, perceptions, and motivations (n = 7) [12–14, 27, 28, 30, 32] and did not report evaluating psychometric properties of included measures. However, only one study mentioned piloting the survey for validation purposes [27]. Ng et al. (2020) mention reviewing their survey for readability statistics, but do not mention other methods of psychometric evaluation [28]. Two studies used measures from United States surveillance systems [30, 31] and one study utilized the Marijuana Effect Expectancies Questionnaire (MEEQ), a previously validated instrument [29].

### Research and practice recommendations

Included studies had numerous recommendations for both future research and practice (Table 3). Surprisingly, only one study mentioned psychometric evaluation of measures for future research [28]. Common areas of future research to address existing knowledge gaps included future studies with a more robust design (e.g., controlling for co-substance use, homogenous populations) and studies examining the etiology of cannabis use among women, including how women’s attitudes, beliefs, and perceptions play a role in the cannabis-related decision making [12–14, 29, 30]. Another recommendation area for future research was examining health care providers’ motives for and influences of cannabis-related recommendations [30, 31].

Most practice recommendations centered on the role of health care providers in preventing potential adverse health outcomes. Specifically, studies recommended that health care providers counsel women about risks of cannabis use to the mother and fetus during pregnancy and postpartum [27, 31, 33, 34]. A single study highlighted that cannabis as a labor analgesic should not be recommended, given absence of safety data [35]. Two studies reiterated the need for public health campaigns that reflected contemporary evidence of risks of prenatal cannabis use [33, 36].

**Table 3:** Research and practice recommendations of included studies

Research	Practice
Further robust studies among homogeneous populations, with stricter inclusion criteria and exclusion of multi-illicit substance use that aim to examine risks of cannabis exposure for both mother and infant during pregnancy and while breastfeeding [13, 14, 26, 28, 30, 31]	Health care providers should educate and counsel women about potential risks of cannabis use in a non-judgement way [14, 28], ensuring coverage of the following topics: - impact of prenatal use on well-being of the fetus [12, 25] - impact of prenatal use on well-being of the mother [27] - avoiding exposure to second-hand cannabis smoke [13]; - adverse health outcomes associated with perinatal cannabis use [25] - social norms and perceived safety [25] - negative effects on fertility [27] - marijuana use while breastfeeding [31]
Research on the etiology of prenatal cannabis use and how beliefs, knowledge, and perceptions influence use [12–14, 29, 30]	Screening and intervention for cannabis use, even in advanced pregnancy stages [14, 25, 30]
Impact of cannabis legalization (both medicinal and recreational) on women’s cannabis use and safety perceptions [12, 31]	Health care providers should be offered training, as part of evidence-based practice programs, to better communicate scientific uncertainty with patients [25]
Research on effective approaches to reduce cannabis use during pregnancy [12]	Breastfeeding mothers should be advised not to use marijuana or marijuana-containing products in any form while breastfeeding [31]
Examination of postpartum cannabis use relapse is warranted [12]	Cannabis use as a labor analgesia should not be recommended without evidence of its safety and efficacy [26]
A further study could address the specific timing of marijuana use on the sexual domains [32]	Health care providers should consider the benefits of counselling on cannabis cessation for patients that are attempting to conceive [27]
Extent of health care provider education, knowledge, and attitudes, and how these may serve as motives for cannabis use recommendations by health care providers [30, 31]	Clear, up-to-date messaging, potentially in the form of public health campaigns, on risks of prenatal cannabis use [12, 28]
Future research should examine effects of cannabis use on female fertility, including if a reduction in use among patients with infertility can improve conception rates [27]	Fertility clinics and government-funded fertility services that typically have eligibility criteria could consider adding cannabis use cessation or abstinence to the list of requirements [27]
Future research could aim to validate the survey items used [28]	A harm-reduction approach may be optimal for women who are unable or unwilling to discontinue using cannabis during pregnancy or while breastfeeding [13]

## Discussion

This is the first review, to our knowledge, to comprehensively examine the breadth of research on measures of antecedents of cannabis use among women of reproductive age. We identified 11 studies reporting on measures of cannabis-related knowledge, attitudes, perceptions, and motivations. We found risk perceptions among pregnant women was the most frequent construct assessed and that most studies were conducted with English-speaking women from hospital or clinic settings. A single study measured the role that health care providers play in women’s cannabis-related decision making. Surprisingly, there were no studies measuring social influences of cannabis use in women. Overall, there was a paucity of evidence, with little to no discussion of psychometric properties of these measures. Thus, we have identified several measurement gaps in this field which future research should aim to address.

In this review, we found a lack of valid, reliable measures to assess antecedents of cannabis use in women of reproductive age, including important maternal health periods, such as the preconception, prenatal, and postpartum periods. With increasing surveillance and research being conducted on women’s cannabis use, the importance of using psychometrically sound measures cannot be understated. Many measures to assess cannabis-related knowledge, perceptions, and motivations in broader, heterogenous populations exist [37–40]. Undoubtedly, future research could look to validate and test for reliability these existing instruments in subpopulations of women. Future research should also prioritize addressing existing measurement-related gaps of cannabis use among women via the creation of psychometrically sound measures to assess antecedents of cannabis use throughout the life span (e.g., adolescence, young adult, preconception, prenatal, postpartum, parenthood), as these may drastically change over time. Importantly, as most prior research was conducted with English-speaking women in health care settings, future research should look how health disparities and health inequities contribute to prenatal cannabis use. As a start, researchers could aim to examine the psychometric properties of instruments or measures included in this review, which would provide a solid foundation from which future research could build.

The lack of available research on measures of antecedents in women of reproductive age poses a challenge to current and future epidemiologic studies that aim to assess cannabis use. Validated and reliable measures of substance use are critical in the success of longitudinal substance use studies, such as the Adolescent Brain Cognitive Development (ABCD) cohort study [41]. Additionally, the lack of evidence on psychometric properties of existing measures is worrisome, as sound psychometric properties are a necessary prerequisite for utilization of any measure [42]. Several included studies used data from nationally-based surveillance systems in the US. However, many of these measures used have yet to be examined for reliability and validity—yet another important area that future research should examine. There has been much qualitative work conducted in this area [15–17, 43]; now researchers should transition to the development and evaluation of quantitative measures. Only after psychometrically sound measures are developed can future work aiming to address associations between antecedents of cannabis use and uptake and continuation of cannabis use begin.

An aim of this scoping review was to elucidate the need for a systematic review on the measures of antecedents of cannabis use among women of reproductive age. Although this is an expanding field, it appears that there is not yet enough empirical evidence to undertake a systematic review. However, researchers could look to conduct a systematic review in this area after this research area has had time to develop and expand. As this is a rapidly growing area of research, we recommend that another scoping review be conducted in 1-2 years and the need for a systematic review be re-evaluated.

### Limitations

There are some limitations of this scoping review. First, we excluded gray literature and studies not published in English, which in turn, could have resulted in failure to identify potentially relevant studies. Second, we utilized date restrictions to capture measures with contemporary relevancy. In doing so, we may have missed in-press or recently published articles yet to be indexed or older articles that may be relevant. Lastly, we attempted to extract psychometric information to include in tabular form in this scoping review but given the lack of psychometric assessment and reporting among included studies, we were unable to do so.

## Conclusions

Amid rapidly changing societal norms and policies regarding cannabis use, those aiming to examine and understand women’s attitudes, perceptions, motivations, and influences of cannabis use uptake and use patterns need measures that are valid, reliable, and easy to use. In this scoping review, however, we found a paucity of evidence in this area, with existing measures limited by breadth, depth, and psychometric soundness, posing a measurement challenge. Ideally, psychometrically sound measures of key constructs should be developed prior to the start of cannabis prevention efforts. Thus, the overarching conclusion of this scoping review is that measurement of women’s cannabis-related knowledge, attitudes, perceptions, motivations, and influences should be a focus of this emerging research agenda.

## Supplementary Information

**Additional file 1.** This file presents the full search strategy used in this scoping review.

## Data Availability

The protocol for this review can be accessed by emailing the corresponding author. All articles included in this review can be accessed online.
