# Supplementary material for: Measuring cannabis-related knowledge, attitudes, perceptions, motivations, and influences among women of reproductive age: a scoping review
Source: BMC Womens Health. 2022 Mar 27;22:95. doi: 10.1186/s12905-022-01673-6 (PMC8961997; doi:10.1186/s12905-022-01673-6)
Supplement: Supplementary file 1 — Additional file 1. This file presents the full search strategy used in this scoping review. [file 12905_2022_1673_MOESM1_ESM.docx]

**Additional File 1: Search Strategy**

Lines 1 to 7 (in black) detail the final search strategy used for this review. The remaining searches in **blue**, detail the full pilot search strategy. The search strategy carried out using a mixture of controlled vocabulary and key words.

| 1 | TI (question* or instrument or tool) OR AB (question* or instrument or tool) |
| --- | --- |
| 2 | TI (women* or maternal) OR TI (prenatal or pregnan*) OR TI (postpartum) OR TI (‘breast feeding’ or breastfeeding) OR AB (women* or maternal) OR AB (prenatal or pregnan*) OR AB (postpartum) OR AB (‘breast feeding’ or breastfeeding) |
| 3 | TI (marijuana OR cannabis) OR AB (marijuana OR cannabis) |
| 4 | 1 AND 2 |
| 5 | 3 AND 4 |
| 6 | TI (perinatal) AND AB (perinatal) |
| 7 | 5 AND 6 |
| 8 | TI (weed or pot or CBD) OR AB (weed or pot or CBD) |
| 9 | 7 AND 8 |
| 10 | TI (survey OR evaluation OR assessment) |
| 11 | 9 AND 10 |
| 12 | 2 AND 12 |
| 13 | TI (weed or pot or CBD) OR AB (weed or pot or CBD) |
